# Mitochondrial Dysfunction in *Ehrlichia canis* Infection

**DOI:** 10.1155/tbed/8787897

**Published:** 2026-05-25

**Authors:** Xishuai Tong, Jing Jiang, Liu Yang, Yunying Liu, Li Chen, Xiaohui Fu, Gengsheng Yu, Naineng Chen, Shuo Tian, Siyang Huang, Shucheng Huang, Jameel Ahmed Buzdar, Xiang Chen, Zongping Liu

**Affiliations:** ^1^ College of Veterinary Medicine, Institutes of Agricultural Science and Technology Development, Joint International Research Laboratory of Agriculture and Agri-Product Safety of Ministry of Education of China, Yangzhou University, Yangzhou, 225009, China, yzu.edu.cn; ^2^ Jiangsu Co-Innovation Center for Prevention and Control of Important Animal Infectious Diseases and Zoonoses, Yangzhou University, Yangzhou, 225009, China, yzu.edu.cn; ^3^ Yangzhou Uni-Bio Pharmaceutical Co., LTD., Yangzhou, 225100, China; ^4^ College of Veterinary Medicine, Henan Agricultural University, Zhengzhou, 450046, China, henau.edu.cn; ^5^ Faculty of Veterinary and Animal Science, Water and Marine Science, Lasbela University of Agriculture, Uthal, Balochistan, Pakistan, luawms.edu.pk; ^6^ College of Bioscience and Biotechnology, Yangzhou University, Yangzhou, 225009, China, yzu.edu.cn

**Keywords:** AMPK, apoptosis, autophagy, dogs, *Ehrlichia canis* (*E. canis*), mitochondrion

## Abstract

Transmitted by tick vectors, *Ehrlichia canis* (*E. canis*) is a Gram‐negative, obligate intracellular bacterium of zoonotic concern that infects both canine and human hosts. Its pathogenesis centers on the targeting of mononuclear phagocytes, where it establishes an intracellular niche by suppressing phagolysosomal fusion and evading immune detection, thereby facilitating its replication. *E. canis* infection also compromises mitochondrial integrity, notably by disrupting the mitochondrial membrane potential (MMP, or ΔΨm), which triggers cellular stress and perturbs critical processes such as autophagy and apoptosis. The energy sensor AMP‐activated protein kinase (AMPK), a central regulator of mitochondrial metabolism and cellular homeostasis, plays a critical role in mediating stress‐responsive pathways, including those governing autophagy and apoptosis. This review examines the interplay between *E. canis* and host mitochondria, with a focus on AMPK‐directed regulation of autophagy and apoptosis during infection. We summarize current knowledge on the mechanisms of mitochondrial dysfunction and AMPK signaling activation, and discuss the dual roles of autophagy and apoptosis in the pathogen’s life cycle and disease progression. By delineating these molecular mechanisms, this review aims to advance the understanding of *E. canis* pathogenesis and inform future strategies for the control of canine monocytic ehrlichiosis (CME).

## 1. Introduction

*Ehrlichia canis* (*E. canis*) is a Gram‐negative, obligate intracellular bacterium that causes canine monocytic ehrlichiosis (CME), a globally distributed tick‐borne disease [1]. The pathogen, *E. canis*, was first identified in 1935 when it was recognized as the cause of canine ehrlichiosis (CE) and shown to be transmitted by the brown dog tick, *Rhipicephalus sanguineus* [2]. As a zoonotic agent, *E. canis* can also infect humans, leading to human monocytotropic ehrlichiosis (HME), but most published cases of HME are caused by *E. chaffeensis*, with only a few publications reporting cases caused by *E. canis* [3, 4]. Despite the reclassification of *Anaplasma* as a genus separate from *Ehrlichia* in 2001, the term “ehrlichiosis” is often still applied to human infections caused by both genera (i.e., *Ehrlichia* and *Anaplasma*). However, it is now recognized that the clinical manifestations and the pathogens themselves are distinct, and human granulocytotropic anaplasmosis (HGA) is caused by *Anaplasma phagocytophilum* (*A. phagocytophilum*) [5].

Recent research has increasingly focused on the mechanisms by which *E. canis* exploits host cells, with particular emphasis on the intricate dynamics of pathogens–host interactions [6–8]. Mitochondria, serving as the cellular powerhouses, are vital for maintaining cellular homeostasis, and their functional integrity is crucial for cell survival. Central to this regulatory network is AMP‐activated protein kinase (AMPK), a master energy sensor that modulates mitochondrial homeostasis, autophagy, and apoptosis in response to intracellular energy levels [9]. Upon infection with *E. canis*, AMPK‐mediated autophagic and apoptotic pathways may be activated to mitigate mitochondrial dysfunction and metabolic stress. This review summarizes the current understanding of the regulation of energy metabolism, autophagy, and apoptosis in *E. canis*‐infected host cells, with a particular emphasis on the role of AMPK in modulating these processes across different stages of infection. Furthermore, it examines how these pathways shape host cell responses to infection, offering theoretical insights into the regulatory mechanisms governing *E. canis*–host interactions.

## 2. The Pathogenesis of *E. canis* and Its Interaction With the Canine Host

### 2.1. Pathogenic Characteristics of *E. canis*

The Gram‐negative, obligate intracellular bacterium *E. canis*, a member of the family *Anaplasmataceae* (order *Rickettsiales*), is a significant pathogen of dogs and humans. This pleomorphic microorganism typically presents as small, coccobacillary entities with a diameter of ~0.5–1.5 μm [6, 10]. Upon staining, *E. canis* exhibits purple coloration with Giemsa and blue or purple with Romanowsky stains. The infection causes CME, a significant clinical disease characterized by the pathogen’s targeting of neutrophils, monocytes, macrophages, and occasionally, vascular endothelial cells [11]. Importantly, the genome of *E. canis* consists of a single circular chromosome that encodes 925 proteins, 40 stable RNAs, and 17 potential pseudogenes, with noncoding sequences accounting for 27% [12]. Additionally, comparative genome analyses with other *Ehrlichia* species (or strains), *E. canis* has significant genetic consistency but also exhibits some unique genomic features. It is worth noting that the authors have obtained an in vitro culture strain of the *E. canis* YZ‐1 strain, and comparative genomic analysis of this strain showed a significant reduction in pathogenicity‐related genes, reflecting an adaptive mechanism that promotes long‐term survival of host cells [13].

### 2.2. The Interaction of *E. canis* With the Host

The primary vector for *E. canis* transmission is the *Rhipicephalus sanguineus* tick, which has a global distribution and is particularly common in tropical and subtropical regions. It is the main vector responsible for the transmission of CE. In addition, other tick species are significant vectors: *Amblyomma americanum* in the Americas and *Haemaphysalis longicornis*, an invasive species native to Asia that has now spread to other regions [7]. Ticks acquire the pathogen by feeding on the blood of infected hosts and transmit *E. canis* to new hosts during subsequent developmental stages. When infected nymphs or adults feed, *E. canis* enters the host through salivary gland secretions at the feeding site, subsequently invading monocytes, macrophages, or other types of cells, thereby inducing infection. After tick transmission, *E. canis* establishes infection by residing within the host’s mononuclear system, which includes the spleen, liver, and lymph nodes [14]. Based on 16S rRNA homology, the genus *Ehrlichia*, is classified into five species: *E. chaffeensis*, *E. ewingii*, *E. ruminantium*, *E. muris*, and *E. canis*. Notably, *E. chaffeensis* and *E. ewingii* can also infect both animals and humans via tick transmission. Furthermore, mosquitoes have been investigated as potential vectors for *E. canis*. Although its DNA has been detected in species such as *Culex quinquefasciatus* and *Aedes aegypti*, their competence in transmitting the pathogen remains unconfirmed [15].

Following phagocytosis during early infection, *Ehrlichia* species are internalized into host cells within endosomal compartments [16]. The pathogen replicates by binary fission and subsequently forms characteristic inclusions observable by transmission electron microscopy (TEM). These structures, known as morulae, exhibit a mulberry‐like morphology [17]. However, their size, staining properties, and detailed ultrastructure vary across *Ehrlichia* species. For instance, *E. canis* produces larger, regularly shaped inclusions in monocytes and macrophages. These vacuoles contain dozens of tightly packed morulae embedded within a fibrous matrix under TEM [18, 19]. After entering the host cell via endocytosis, *E. canis* remains aggregated inside vacuoles that avoid fusion with lysosomes, thereby facilitating rapid intracellular replication within mononuclear phagocytes [20].

### 2.3. *E. canis* Infection in Dogs

As a specialized intracellular parasite, *E. canis* primarily targets canine monocytes and macrophages. Following invasion, *E. canis* binds to the intracellular structure to hijack host signaling molecules, facilitating its replication, proliferation, and maturation, while concurrently scavenging host nutrients to sustain its infectious cycle [21]. This intracellular lifestyle enables *E. canis* to evade immune detection and phagocytosis, ultimately triggering mitochondrial damage, oxidative stress, and a cascade of downstream cellular and systemic responses [22]. Furthermore, *E. canis* infection impairs lymphocyte mitosis and suppresses antibody production, leading to broad immunosuppression. Nevertheless, the precise mechanism through which *E. canis* avoids lysosomal degradation, thereby disrupting phagocytic function and dampening immune responses, remains to be fully elucidated [23]. Of practical relevance, *E. canis* can be maintained and propagated in the canine macrophage‐derived renal malignant histiocytosis cells (DH82 cells), an established in vitro model derived from malignant histiocytosis. Infection of DH82 cells with *E. canis* provides a valuable experimental system for dissecting the dynamic interplay between this pathogen and its host [22].

In dogs, *E. canis* infection typically progresses into three stages: the first stage, the acute phase, lasts for 2–4 weeks following transmission by infected ticks (incubation period: 8–20 days). The affected dogs exhibit symptoms such as fever, depression, dyspnea, anorexia, and mild weight loss. Laboratory tests have found thrombocytopenia and leukopenia. The subclinical phase persists for 6–20 weeks or even for several years; during this phase, dogs remain asymptomatic but are accompanied by mild thrombocytopenia. Importantly, in the chronic phase, dogs exhibit features such as bleeding, epithelial swelling, and edema, accompanied by mild clinical symptoms resembling those in the acute phase. However, this stage may be more complicated due to repeated infections by other microorganisms [24]. Prognosis is poor for most dogs due to bone marrow hypoplasia, leading to progressive clinical deterioration [25]. Notably, dogs infected with *E. canis* may remain carriers for life, even if they receive treatment with antibiotics such as doxycycline [26].

Humans can also become accidentally infected with *E. canis* and exhibit latent asymptomatic or latent infection [27]. Similarly, infection in humans progresses through acute, subclinical, and clinical or chronic stage. During the acute phase, *E. canis* proliferates in leukocytes, presenting as nonspecific symptoms that gradually transition to the subclinical stages. Within 1–2 weeks of postinfection, patients may develop symptoms such as fever, headache, myalgia, and general weakness and may occur, manifested as intumescence of the spleen and liver and glandular disease. Hematological abnormalities such as anemia, thrombocytopenia, and leukopenia are frequently observed. Moreover, ~40% of patients develop clinical multisystemic manifestations, such as cough, pharyngitis, lymphadenopathy, diarrhea, vomiting, abdominal pain, and altered mental status. In addition, *E. canis* infection can also lead to severe bleeding, neurological disorders, and ocular pathologies, such as retinal vascular tortuosity, subretinal hemorrhage, etc. [7, 27]. In some special cases, patients may also present clinical symptoms, such as chills, rash, arthralgia, and bone pain in addition to the aforementioned symptoms [28]. In severe cases, *E. canis* can lead to multiple‐organ failure and even death. Beyond dogs and humans, *E. canis* also infects other animals such as deer, wolves, dairy cattle, and goats, and exhibits different clinical symptoms and pathogenicity [29, 30].

## 3. *E. canis* Infection Modulates Mitochondrial Autophagy and Apoptosis at the Host–Pathogen Interface

### 3.1. The Host–Pathogen Interface: *E. canis* Infection and Mitophagy

Autophagy is a process of self‐degradation in eukaryotic cells, mainly through the lysosome‐mediated dissolution mechanism to eliminate damaged organelles, misfolded proteins, and other large macromolecules, to maintain cellular homeostasis, including macroautophagy, microautophagy, and chaperone‐mediated autophagy (CMA) [31]. In response to macroautophagy, the *E. canis* YZ‐1 strain infected with DH82 cells or Vero cells promotes the autophagy by increasing the expression of autophagy marker microtubule‐associated protein 1A/1B light chain 3 (LC3) and reducing the expression of p62. However, the use of lysosome inhibitors increased the growth of the *E. canis* YZ‐1 strain in host cells, indicating that *E. canis* YZ‐1 can utilize host cell autophagy to obtain nutrients for amplification or reproduction [21]. *E. canis* YZ‐1 infection forms endosomes that do not bind to lysosomes, thereby evading the host cell’s clearance of foreign pathogens [21]. Beyond this nonselective degradation, cells employ selective autophagy to target specific cargo. Mitophagy, the selective autophagic elimination of mitochondria, is of particular interest in the context of intracellular bacterial infections that impact mitochondrial health. In addition, selective autophagy is a self‐degradation mechanism in which cells maintain cellular homeostasis by specifically recognizing and degrading specific cellular components, such as damaged mitochondria, endoplasmic reticulum, and lipid droplets, and it can be divided into mitophagy, lipophagy, and reticulophagy, etc. It is well established that mitophagy is the most extensively characterized autophagic pathway [32]. Mitophagy is a selective autophagic mechanism that mainly participates in regulating the clearance of mitochondrial “waste” in cells and can also clear or restrict pathogen proliferation to maintain the normal mitochondrial function [33].

**Figure 1:**
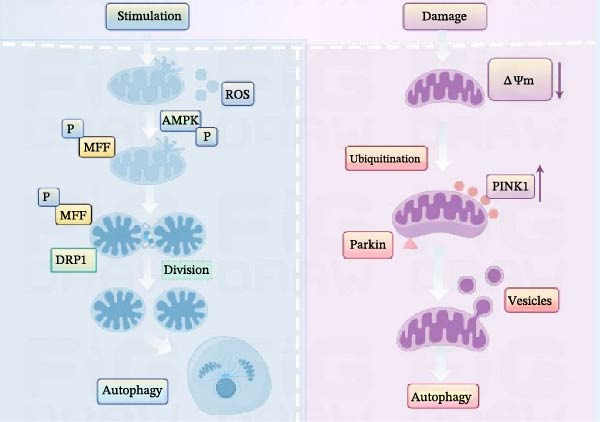
Schematic diagram of the mitochondrial signaling pathway triggered by stimulation. Stimulation (not confined to pathogens such as *E. canis*) induces phosphorylation (P) events that activate mitochondrial MFF, leading to recruitment of DRP1 and subsequent mitochondrial division. This process is linked to autophagy. Stimulation also increases ROS levels and activates AMPK, and phosphorylated AMPK may participate in the regulation. Moreover, stimulation promotes the release and changes of a wide array of damage‐associated molecular patterns (DAMPs) and related processes and molecules (ΔΨm, ubiquitination, Parkin, PINK1, and the vesicle formation and increases), which likely contribute to the inflammatory response or further cellular signaling. The white, light‐colored thick arrows in the figure indicate the direction of event progression, the light‐purple arrow indicates an increase (or rise), and the single letter “P” represents phosphorylation.

However, there is not much direct evidence to suggest how *E. canis* specifically affects mitochondrial function in canine cells, such as DH82 cells. Correspondingly, findings from other studies on the interaction between *E. canis*‐like pathogens and mitochondria provide useful references, including that various species of *Ehrlichia* may exert important regulatory effects on host cell mitochondria. Liu et al. [34] reported that mitochondria are specifically recruited by *E. chaffeensis*‐infected DH82 cells, and *E. chaffeensis* inhibits mitochondrial metabolism by repressing DNA synthesis to maintain the normal mitochondrial membrane potential. *E. ewingii* is the only *Ehrlichia* species to infect canine neutrophils and can delay canine neutrophil apoptosis in vitro culture by stabilizing mitochondrial integrity (by maintaining mitochondrial membrane potential) of host neutrophils [35]. During pathogen infection, mitophagy reduces the damaged mitochondria, the accumulation of reactive oxygen species (ROS), and oxidative stress, thereby protecting host cells from further damage. This is crucial for maintaining intercellular energy metabolism and reducing the pathogen’s ability to destroy the host under infection conditions [36]. Research has shown that pathogen infection of host cells can lead to mitochondrial dysfunction and achieve self‐replication by “combating” mitophagy, even resulting in immune evasion [37]. Mitophagy typically involves the PTEN‐induced putative kinase 1 (PINK1)–Parkin‐dependent ubiquitin pathway and receptor‐mediated non‐ubiquitin pathways. When mitochondrial membrane potential is damaged during the PINK1–Parkin‐dependent ubiquitin pathway, the depolarization of the damaged mitochondria leads to the accumulation of PINK1 protein on the outer membrane of the mitochondria and activation of Parkin, which undergoes a change in spatial conformation, is recognized by autophagosomes through ubiquitination, and leads to the degradation of “waste” (Figure 1) [38]. A study on *E. japonica* found that it caused severe and fatal rickettsial disease in immunocompetent mice mimicking severe HME, infecting and replicating in hepatocytes. *E. japonica* infection in murine primary hepatocytes caused the reduction of PINK1 and Parkin expression at mRNA and protein levels [39].

### 3.2. Role of Pathogen Infection in Host Cell Apoptosis

Apoptosis is an active, programmed cell death process and a critical phenomenon in cellular biology, including the programmed degradation of DNA, chromatin condensation, reduction and fragmentation of cell volume, nuclear pyknosis, and formation of apoptotic bodies [40]. Pathogen infection leads to the inability to restore cell function or damage of host cell exceeding a certain threshold, mitophagy is insufficient to maintain or restore cell survival, and it fails to effectively remove the injurious biomolecules and unfolded proteins, etc., the cell will initiate apoptosis signals and promote apoptosis. The apoptotic signals are stimulated by certain signals such as DNA damage, deprivation of growth factor, and mitotic arrest, to activate proapoptotic proteins Bcl‐2 associated X‐protein (Bax) and Bcl‐2 antagonist/killer (Bak), and determine whether cells choose autophagy or apoptosis by regulating mitochondrial outer membrane permeabilization (MOMP) and signaling molecules in autophagy pathways [41]. Rikihisa [42] study reported that *E. chaffeensis* outer‐membrane invasion protein EtpE, binds to its cognate receptor DNase X on the host‐cell plasma membrane, triggers the formation of actin polymerization and filopodia, and catalyzes the production of microbicidal ROS by blocking the activation of nicotinamide adenine dinucleotide phosphate (NADPH) oxidases. Subsequently, the *Ehrlichia* translocated factor‐1 (Etf‐1) is one of the components of the type IV secretion system (T4SS) of *E. chaffeensis*, and also localizes to the mitochondria of host cells to inhibit mitochondria‐mediated apoptosis [43].

The pathways of apoptosis activities by pathogens in host cells, including the endogenous pathways mediated by mitochondria and the exogenous pathways mediated by death receptor. The increase of outer membrane permeability of mitochondria and the release of proapoptotic factors such as cytochrome C during the endogenous pathway mediated by mitochondria, activating the caspase cascades and promoting cell apoptosis [38]. Caspase cascades of downstream executing caspases are initiated through mutual activation under the induction of apoptosis signals and mainly involve two pathways: one is the exogenous death receptor pathway, mainly involving the activation of caspase‐8, and the other pathway is the mitochondrial pathway, which mainly involves the activation of caspase‐9. The cleavage and activation of executive caspases are performed by caspase‐9, which further cleave proteins within the cell, leading to structural damage of cells and even death [44]. Da Silva et al. [45] reported that dogs infected with *E. canis* will experience redox imbalance by increasing nitrite/nitrate (NOx) concentrations, lipid peroxidation (TBARS), advanced oxidized protein products (AOPP), and glutathione reductase (GR) activity. Additionally, dogs naturally infected with *E. canis* exhibit changes in iron metabolism and the oxidative environment [46]. Like the impact of *E. canis* infection on the mitochondrial functional homeostasis of host cells, there is still a lack of direct experimental evidence and specific data. Research on members of the *Ehrlichia* family suggests that mitochondrial dysfunction in host cells may be closely related to the development of diseases. Hepatocytes infected with *E. japonica* showed elevated levels of ROS, changes in mitochondrial membrane potential, and abnormal mitochondrial morphology (swollen matrices and cristae damage), indicating that virulent *Ehrlichia* causes mitochondrial dysfunction. Correspondingly, the blockade of autophagy flux and mitochondrial autophagy may be partially responsible for the observed mitochondrial damage in infected cells. These events may lead to a decrease in cell survival rate in mice and humans after severe *Ehrlichia* infection and exacerbate cell death in the form of apoptosis and necrosis [39, 47].

However, mitophagy can also inhibit the release of apoptotic signaling by removing damaged mitochondria to prevent excessive apoptosis of cells [48]. Therefore, this balance mechanism is particularly important in controlling infections in host cells by pathogens such as viruses, bacteria, and parasites, but actively inducing host cell apoptosis under certain conditions can limit the replication and spread of pathogens [49]. The roles of mitophagy and apoptosis in pathogen infection are not independent but rather mutually regulated and influenced. However, their interrelationships are complex, such as mitophagy can inhibit apoptosis to help cell survival by eliminating pathogens, and excessive mitophagy can further trigger cell death [48]. Perhaps it is necessary to use existing methods or new technologies to test related species in canine models in the future, such as transcriptomic or proteomic analysis of *Ehrlichia* infected DH82 cells or mitochondrial stress measurement in experimentally infected dogs.

### 3.3. Coordination of Autophagy and Apoptosis During *E. canis* Like Pathogen Infection

The regulatory mechanisms of autophagy and apoptosis induced by pathogens similar to *E. canis* may also exist, such as *A. phagocytophilum*, which is an intangible body parasite in host cells and mainly infects host neutrophils. To establish intracellular survival, *A. phagocytophilum* creates a specialized membrane‐bound parasitophorous vacuole by subverting vesicular trafficking, thereby evading lysosomal degradation. Concurrently, *A. phagocytophilum* promotes cell survival by inhibiting apoptosis and activating prosurvival signaling pathways, including phosphatidylinositol 3‐kinase (PI3K)/protein kinase B (AKT) and nuclear factor‐kappa B (NF‐κB) pathways [50–52]. Research has found that the T4SS of *A. phagocytophilum* is crucial for the survival and replication of host cells by inducing autophagy to obtain nutrients and inhibiting apoptosis of host cells [53]. Similarly, HGA is also caused by *A. phagocytophilum* infection of peripheral blood neutrophils,and is a tick‐borne disease characterized by fever accompanied by the decrease in leukopenia and platelets and damage to multiple organ functions [54–56]. Meanwhile, a study has shown that *A. phagocytophilum* infection leads to oxidative burst, apoptosis, and inhibition of phagocytic function in neutrophils, activating degranulation and cytokine secretion, which are partially mediated by changes in host transcription levels [57]. In addition, *A. phagocytophilum* is also transmitted by ticks and can infect different tissues such as the midgut and salivary glands, thereby regulating the apoptosis pathway. For example, in the midgut, *A. phagocytophilum* suppresses the apoptosis of host cells by activating the Janus kinase (JAK) / signal transducers and activators of transcription (STAT) pathway, which facilitates infection, while in the salivary glands, *A. phagocytophilum* inhibits endogenous apoptosis pathways by downregulating porin expression and can activate exogenous apoptosis pathways to limit infection, indicating that the regulation of *A. phagocytohilum* varies in different tissues [58].

*Neorickettsia sennetsu* is also an obligate intracellular pathogen, commonly believed to be infected through human consumption of raw fish slices, causing sennetsu fever but not being fatal [59]. Dysregulation of host phospholipid metabolism serves as a key mechanism by which *N. sennetsu* suppresses both apoptosis and autophagy. In addition, *Rickettsia* (including *E. canis*) blocks apoptosis defense strategies of host cells, prolongs the lifespan of infected cells, promotes its replication, and then spreads to other host cells [60]. *Rickettsia typhi* interacts with the PI3K signaling of host cells via Risk1, which targets the “Rab5–EEA1–phosphatidylinositol 3‐phosphate (PI3P)” axis, promoting its “escape,” while R. typhus undergoes ubiquitination and induces host cell autophagy. On the contrary, the fusion and maturation of autophagic lysosomes in host cells are disrupted for cell survival, which facilitates the replication of *R. typhi* in the cytoplasm of host cells and prevents host cell apoptosis [61]. Correspondingly, the *Rickettsia* spotted fever groups (such as *R. rickettsii* and *R. conorii*) and typhoid fever group (such as *R. typhi* and *R. prowazekii*) disrupt different processes within host cells, including membrane dynamics, actin cytoskeleton remodeling, phosphoinositide metabolism, intracellular transport, and immune defense, promoting host colonization and intercellular transmission through secreted effectors (virulence factors) [62]. However, there are relatively few reports on the autophagy and apoptosis regulation mechanisms of *E. canis*‐infected host cells, which poses a challenge for further exploration of *E. canis* infection in host cells and its pathways of action.

## 4. AMPK Orchestrates Host Cell Responses to *E. canis* Infection

### 4.1. AMPK‐Mediated Autophagy Modulates Cellular Energy Metabolism

The AMPK is a heterotrimeric protein and is composed of one catalytic α‐subunit and two regulatory structures of β and γ subunits [63]. AMPK, as the regulatory center of cellular energy metabolism, not only maintains cellular energy equilibrium under normal physiological conditions but also plays a critical regulatory role in various stresses or pathogen infections. AMPK can be activated under glucose starvation by directly perceiving the glycolytic intermediate fructose‐1,6‐bisphosphate (FBP), and then recruiting AMPK into the “supercomplex” of the lysosomal membrane [64]. Similarly, heat stress can activate AMPK by increasing mitochondrial ROS, reducing blood oxygen levels, and altering DNA integrity [65]. For energy metabolism, activated AMPK enhances mitochondrial oxidative metabolism and glucose uptake to provide ATP to counteract metabolic stress and facilitate survival [66]. In addition, AMPK directly or indirectly activates autophagy by stimulating UNC‐51‐like autophagy activating kinase 1 (ULK1) and inhibiting the mammalian target of rapamycin (mTOR) pathway [67].

AMPK promotes mitochondrial fission by phosphorylating mitochondrial fission factor (MFF), which is the main receptor for mitochondrial dynamic‐related protein 1 (DRP1) located on the outer membrane of mitochondria and also serves as a substrate at phosphorylation sites of Ser155 and Ser173 for AMPK‐mediated mitochondrial contraction during fission, increasing the number of damaged mitochondria and making them easily recognized and removed by autophagosomes [68]. As mentioned above, mitophagy involves two types: the ubiquitin‐dependent pathway (mediated by PINK1 and Parkin) and the ubiquitin‐independent pathway (involving mitophagy receptors). However, AMPK balances the quantity and quality of mitochondria by regulating their functions in mitophagy. For example, AMPK activates autophagy by phosphorylating ULK1 at Ser555, while mTOR suppresses autophagy by phosphorylating ULK1 at Ser757 (Ser758 in this study), causing AMPK to dissociate from ULK1 [69, 70].

### 4.2. The Role of AMPK Signaling in Coordinating Host Cell Responses to *E. canis* Infection

As mentioned above, the role of AMPK signaling in the host response to *E. canis* is underexplored. Lin et al. [71] reported that *E. chaffeensis*‐stimulated autophagy is independent of the general cellular ubiquitination pathways involving mTOR and AMPK. In addition, *E. canis* can trigger immune responses or programmed cell death after infection of host cells, mainly infecting the host mononuclear/phagocytic cell system and regulating multiple complex immune and molecular mechanisms. *E. canis* infection can significantly reduce the expression of major histocompatibility complex II (MHCII) on the cell surface and inhibit antigen presentation and impair the innate immune response in host cell DH82 for facilitating its survival in host cells [72]. Nambooppha et al. [73] found that a large number of morulae were observed in DH82 cells in the early stage of *E. canis* infection, which were implanted in the cytoplasm and significantly reduced the levels of *IFNG* (a protein coding gene of interferon‐γ [IFN‐γ]), *interleukin-10* (*IL-10*), *IL-12B*, and *IL-13*. The inhibiting mechanism of *E. canis* infection on DH82 cells was explored using the model of glycoprotein GP19_4–13_ to induce superimmune serum in rabbits, showing that GP19_4–43_ serum reduced *E. canis* infection on the third day and significantly upregulated *IFNG* levels [73]. However, GP19_4–43_ serum can promote the levels of IFN‐γ in DH82 cells to enhance the immune response induced by *E. canis*, but the specific regulatory mechanism still needs to be further demonstrated [73]. The apoptosis response of DH82 cells induced by *E. canis* infection involves complex immune suppression and molecular regulatory mechanisms, which not only affect the host’s immune response but may also provide favorable conditions for the survival of *E. canis*. However, the mechanistic understanding of how MHC‐mediated mitochondrial dysfunction engages with these immune pathways remains limited.

**Figure 2:**
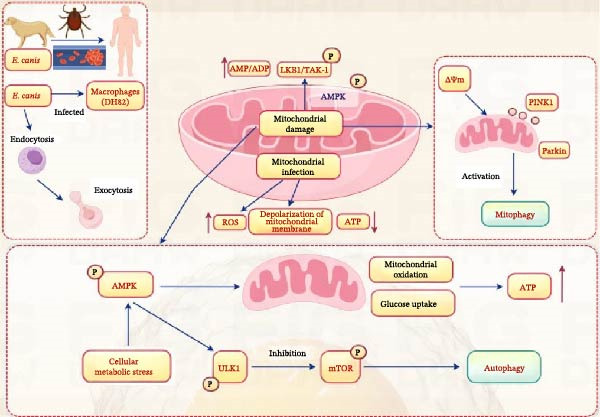
Schematic illustration of *E. canis* infection in macrophages (DH82 cells) and the subsequent mitochondrial and metabolic signaling events. *E. canis* enters host cells via endocytosis and is released through exocytosis. Mechanically, *E. canis* infection causes mitochondrial damage, leading to depolarization of the ΔΨm, increased ROS production, and ATP depletion. These changes activate the PINK1/Parkin pathway, triggering mitophagy. Concurrently, cellular metabolic stress mediated by LKB1/TAK‐1 and an increased ADP/ATP ratio activates AMPK. Subsequently, activated AMPK inhibits mTOR and phosphorylates ULK1, thereby promoting autophagy and glucose uptake. These coordinated mitochondrial and metabolic adaptations facilitate intracellular bacterial survival and contribute to the pathogenesis of *E. canis* infection. Collectively, *E. canis* hijacks mitochondrial dynamics and energy sensing pathways to promote host metabolic reprogramming and autophagic adaptations, creating a permissive niche for *E. canis* persistence.

*E. canis* is different from other Gram‐negative pathogens, mainly due to its cell wall being very flexible because of absence of peptidoglycan and lipopolysaccharides (LPS), which helps it evade immune attacks from the host immune system. This immune suppression occurs alongside significant metabolic stress within the host cell. As *E. canis* lacks key biosynthetic pathways and relies on host amino acids for energy, the resulting nutrient deprivation is a potent stimulus for AMPK [74]. Therefore, the availability of nutrients can also play a role during the infection of *Ehrlichia*. Infection with *Ehrlichia* can cause changes in cellular metabolism in host cells, including alterations in glucose and fatty acid metabolism, which can affect the availability of nutrients in cellular processes. Research has shown that nutritional starvation has an impact on the mTOR complex 1 (mTORC1) and AMPK, which are key regulatory factors in cellular metabolism [75]. However, there is limited data on the role of AMPK in *E. canis* infection of host cells, and future research is needed to further understand the relationship between *E. canis* and AMPK‐mediated energy metabolism in host cells (Figure 2).

## 5. Conclusions and Future Perspectives

AMPK is a master regulator at the intersection of metabolism, mitophagy, and apoptosis, making it a prime target for *E. canis* manipulation. To move the field forward, several critical, testable questions emerge: (1) Does *E. canis* infection actively activate or inhibit AMPK, and how does this change over the course of infection? (2) Using pharmacological agents (metformin, AICAR, and compound C) in the DH82 infection model, can we directly test whether AMPK activity is necessary or sufficient for bacterial survival and the observed changes in mitophagy/apoptosis? (3) Does *E. canis* secrete a T4SS effector, analogous to *A. phagocytophilum’s* Ats‐1, that directly targets mitochondrial function or the AMPK pathway? Answering these questions will transform our understanding from passive description to mechanistic insight.

Beyond these specific questions, we speculate that the mechanism of AMPK in *E. canis* infection may involve multiple aspects based on the mechanisms of action of other *Rickettsia*, including autophagy, metabolic regulation, immune response, and apoptosis. Especially, how *E. canis* infects host cell mitochondria and how mitochondria‐mediated autophagy and apoptosis exert regulatory effects remain poorly understood. Advances in modern molecular technologies will enable research to move beyond general descriptions, facilitating in‐depth investigations into specific relationships and mechanisms of action. Key areas of focus include the therapeutic challenges associated with targeting host metabolism, the integration of mitochondrial stress signals with innate immune pathways (e.g., cyclic GMP‐AMP synthase [cGAS]‐stimulator of interferon genes [STING] or NACHT, LRR, and PYD domains‐containing protein 3 [NLRP3]), and the development of testable hypotheses regarding how *E. canis* and its close relatives uniquely interface with these systems. Given the importance of canine *E. canis* as a pathogen, ongoing efforts to provide and continuously update our understanding of its pathogenic mechanisms are essential for informing future therapeutic and preventive strategies.

AMPK, as a potential energy metabolism target, may play a key role in controlling the pathological process of *E. canis* infection. Although pharmacological agents such as metformin, compound C, and AICAR are well‐established tools for studying AMPK pathways, the role of AMPK in host cell responses to *E. canis* infection remains entirely unknown. Furthermore, it is unclear whether *E. canis* exploits mitochondrial and AMPK regulation as an immune evasion strategy to persist within the host, a hypothesis that awaits experimental validation. However, the specific regulatory mechanisms of the AMPK signaling pathway in different pathological states still need further investigation, and future research should delve into the specific regulatory mechanisms of AMPK on host cell mitochondria and its mediation of autophagy and apoptosis during *E. canis* infection. In addition, further revealing the interactions between AMPK and other signaling pathways such as mTOR and mitogen‐activated protein kinase (MAPK) will help to comprehensively understand the response mechanism of host cells to pathogen infections, provide a theoretical basis for developing new therapeutic strategies, and explore their potential application value in anti‐infective therapy.

In summary, the crucial role of AMPK in mitophagy and apoptosis provides a new perspective for studying *E. canis* infection, and future research will help reveal more details about host–pathogen interactions.

**Nomenclature**
AKT: Protein kinase B
AMPK: AMP‐activated protein kinase
AOPP: Advanced oxidized protein products
Bak: Bcl‐2 antagonist/killer
Bax: Bcl‐2‐associated X protein
CE: Canine ehrlichiosis
cGAS: Cyclic GMP–AMP synthase
CMA: Chaperone‐mediated autophagy
CME: Canine monocytic ehrlichiosis
DRP1: Dynamic‐related protein 1
Etf‐1: Ehrlichia translocated factor 1
FBP: Fructose‐1,6‐bisphosphate
GR: Glutathione reductase
HGA: Human granulocytotropic anaplasmosis
HME: Human monocytotropic ehrlichiosis
IFN‐γ: Interferon‐γ
IL‐10: Interleukin‐10
LC3: Microtubule‐associated protein 1A/1B light chain 3
LPS: Lipopolysaccharide
MAPK: Mitogen‐activated protein kinase
MFF: Mitochondrial fission factor
MHCII: Major histocompatibility complex II
MMP (ΔΨm): Mitochondrial membrane potential
MOMP: Mitochondrial outer membrane permeabilization
mTOR: Mammalian target of rapamycin
mTORC1: Mammalian target of rapamycin complex 1
NADPH: Nicotinamide adenine dinucleotide phosphate
NF‐κB: Nuclear factor kappa B
NLRP3: NACHT, LRR, and PYD domains‐containing protein 3
NOx: Nitrite/nitrate
PI3K: Phosphatidylinositol 3‐kinase
PI3P: Phosphatidylinositol 3‐phosphate
PINK1: PTEN‐induced putative kinase 1
ROS: Reactive oxygen species
TEM: Transmission electron microscopy
TBARS: Lipid peroxidation
ULK1: UNC‐51‐like autophagy activating kinase 1.

## Author Contributions

**Xishuai Tong**: data curation, visualization, writing – review and editing. **Jing Jiang:** data curation, formal analysis, resources, writing – original draft, writing – review and editing. **Liu Yang and Yunying Liu**: writing – original draft, writing – review and editing. **Li Chen**: resources, writing – review and editing. **Xiaohui Fu:** formal analysis, writing – review and editing. **Gengsheng Yu:** formal analysis. **Naineng Chen and Shuo Tian:** methodology. **Siyang Huang:** methodology, validation. **Shucheng Huang:** validation. **Jameel Ahmed Buzdar:** writing – original draft. **Xiang Chen:** writing – review and editing. **Zongping Liu:** data curation, supervision, validation, visualization, writing – original draft.

## Funding

This work was supported by the 111 Project D18007, a project funded by the Priority Academic Program Development of Jiangsu Higher Education Institutions (PAPD), the Science and Technology Innovation Special Project of Joint International Research Laboratory of Agriculture and Agri‐Product Safety of The Ministry of Education of China (Innovation and Entrepreneurship Training Program for College Students of Yangzhou University), and the Qing Lan Project of Yangzhou University.

## Ethics Statement

The authors have nothing to report.

## Consent

The authors have nothing to report.

## Conflicts of Interest

The authors declare no conflicts of interest.

## Data Availability

No datasets were generated or analyzed in this study.
